# Uncovering economic impacts and dynamics of European energy policy: Evidence from DEMATEL, panel data, and cluster analysis

**DOI:** 10.1371/journal.pone.0322525

**Published:** 2025-11-04

**Authors:** Aldona Kluczek, Agnieszka Woźniak, Patrycja Żegleń

**Affiliations:** 1 Warsaw University of Technology, Faculty of Mechanical and Industrial Engineering, Warsaw, Poland; 2 State University of Applied Sciences in Krosno, Krosno, Poland; 3 University of Rzeszów, Faculty of Economics and Finance, Rzeszów, Poland; Czestochowa University of Technology: Politechnika Czestochowska, POLAND

## Abstract

This study analyzes the relationship of national diversity in energy policies between economic outcomes amid changing energy prices, climate regulations, and technological innovations across European countries. Using DEMATEL, panel data econometric models, and cluster analysis, the research three interrelated questions: whether environmental regulations can simultaneously support economic performance and innovation; how energy prices and climate policies affect inflation, economic growth, and unemployment; and whether a hybrid causal framework can reveal deeper feedback dynamics across policy, innovation, and macroeconomic variables, The results identify significant relationships between technological innovations, energy prices, climate regulations, and inflation. The analysis reveals that energy prices influence R&D expenditure, with both positive and negative effects depending on timing. Climate regulations and inflation also significantly impact technological innovations. The findings emphasize the need for strategic planning and investment in technology to manage energy prices and climate policies effectively. The study suggests that stable, innovation-oriented regulatory frameworks are more effective than short-term interventions such as subsidies or price caps in promoting green technologies, reducing economic volatility, and supporting the transition to sustainable energy systems. Limitations include the exclusion of variables such as institutional quality, consumer behavior, and readiness for energy storage infrastructure.

Further research with extended time series and localized data is recommended to deepen understanding and support resilient energy policy development.

## 1. Introduction

Understanding the complex mechanisms behind energy policy formation and its economic impacts is increasingly vital, as Europe faces heightened challenges related to climate imperatives, energy market volatility, and innovation demands. Contemporary energy systems are not only the backbone of industrialized economies [1], but also highly sensitive to regulatory interventions, technological disruptions, and geopolitical events [2–5]. Europe’s heterogeneous energy policy landscape reflects deep-rooted socioeconomic and infrastructural differences [6], which have intensified in response to recent supply crises and shifts in environmental regulation [7–9]. Despite extensive research on energy economics, comparative insights into how energy policies, prices, climate regulations, and innovation interact across EU economies remain limited. Prior studies have typically addressed isolated factors, such as carbon pricing, renewable energy deployment, or infrastructure modernization [10–12], with few exploring the cross-national causal structures of these relationships.

In Central and Eastern European (CEE), for instance, inflation been tightly linked to energy price fluctuations [13], yet countries vary sharply in their capacity to reduce import dependency. Estonia has bolstered renewables, while others remain vulnerable to international price fluctuations. While Estonia has bolstered renewables, others remain vulnerable to international shocks. The divergence in energy strategies, e.g., Hungary’s household energy price reduction of 41% (2010–2021) compared to price increases elsewhere, underscores the need for systemic and comparative analysis [14].

Moreover, most studies focus on single-country cases or renewable-intensive economies [15,16], overlooking broader economic and policy interconnections. Yet EU countries must simultaneously pursue decarbonization and economic resilience. As the EU pushes for integrated energy governance, there is a need to assess policy interactions holistically through the combined lens of climate regulation, energy pricing, innovation, and macroeconomic stability [17].

To addresses this gap, the present study examines the causal architecture of European energy policy across diverse national contexts, using DEMATEL and panel econometric methods to disentangle the interlinkages among policy variables, including climate regulations, energy prices, and R&D expenditures. Unlike studies focusing narrowly on renewables or national transitions, our approach enables cross-country comparisons and captures feedback dynamics under conditions of policy uncertainty. The goal is to reveal not only direct effects, but also deeper feedback dynamics that influence strategic energy planning across Europe.

Moreover, to ground the analysis in a theoretical framework, the analysis draws on Porter’s hypothesis which posits that well-designed environmental regulations can stimulate countries’ innovativeness [18]. While influential in shaping ecological policy, this view remains controversial. Many researchers [19] argue that environmental regulations can burden countries financially and administratively, while others [20,21] emphasize that effects depend on factors such as industry, type of regulations, and national context. Nevertheless, the hypothesis has informed policies such as energy efficiency regulation and highlights the role of policy in driving innovation and competitiveness.

In addition, the literature considers the role of clusters [22–25] – geographic concentrations of interconnected companies and institutions – as mechanisms that enhance competitiveness and facilitate technological adaptation under environmental constraints [26]. Accordingly, the clustering method was used to justify the grouping of countries for comparison purposes.

In contrast to previous research emphasizing the benefits of renewables alone [15,16], this study examines broader regional differences in policy responses and their long-term economic effects under policy uncertainty. It also explores relationships between energy price fluctuations, climate regulations (GH emissions), and technological innovations (R&D), and how they interact with inflation (HICP), unemployment (UE), and economic growth (GDP).

Using panel data for the period 2012–2022, the study provides new evidence on long-term policy effectiveness and interactions, particularly in a context of economic uncertainty and regulatory divergence. While the EU aims to harmonize energy systems, national policy differences continue to affect integration, innovation and sustainability transition goals. By offering a multi-country, casual analysis, this paper supports evidence-based policymaking for resilient and sustainable energy transitions.

Grounded in the Porter Hypothesis and the energy-growth nexus literature, this study explores three central questions: (1) Can environmental regulations be both economically viable and innovation-enabling? (2) What are relations between energy prices and climate policy, technological innovations and macroeconomic indicators such as inflation, economic growth, and unemployment in the EU? (3) How can a hybrid causal approach enhance the understanding of causal linkages among energy prices, climate policy, innovation, and economic performance under policy uncertainty?

These questions shape the structure of the analysis and the design of the methodological framework.

By examining a broad spectrum of policy types and technological capacities, the study captures how national policy diversity shapes economic outcomes across the EU. Finally, the study is not limited to countries with high shares of renewable energy [15] but examines a broader spectrum of policy types and levels of technological advancement, considering regional variations in the relationship between policies and economic performance.

## 2. Literature review

Global attention is increasingly focused on how energy use impacts international trade and economic growth, with EU member states adopting diverse energy policies based on their priorities, resources, and political contexts. These range from investments in renewables and efficiency measures to reliance on fossil fuels and nuclear power.

While the European Energy Union aims to deliver secure, sustainable, and affordable energy, national policies must align with the broader EU regulations under the Paris Agreement (2015) and the European Green Deal (2019). However, there is a research gap in systematically analyzing how policy heterogeneity affects the EU’s economic landscape, as most studies focus on individual countries or specific policy aspects rather the broader EU-wide dynamics [27].

To address this, Supplementary Material S1 Table presents a comparative overview of ten energy-importing countries (Germany, France, Sweden, Poland, The Netherlands, Spain, Denmark, Belgium, Italy, and Norway), representing Central, Eastern, Western, Southern, and Scandinavian Europe. These countries were selected due to their rising energy costs, climate regulation intensity, and advancements in energy technologies, as well as their GDP growth over the last decade, ensuring a diverse representation of policy approaches and their economic implications. The table consolidates data and evidence on national energy policy approaches, their economic implications, and related drivers such as inflation, innovation, and regulatory response [28–32].

The three main factors (energy prices, climate regulations, and technological innovations) are deeply interconnected. They collectively shape production costs, market dynamics, investment decisions, and macroeconomic indicators such as economic growth, inflation, and unemployment. Rising energy prices increase inflation, production costs, and reduce competitiveness, potentially dampening demand [33–35]. Climate regulations, such as carbon pricing and emissions standards, reduce environmental impact but may raise compliance costs and investment uncertainties [36]. Meanwhile, technological innovations enhance productivity, drive demand, boost economic resilience, and support structural transformation [37]. Understanding these multidimensional interactions is vital for developing adaptive and resilient energy policy frameworks and sustainable economy across Europe.

For policymakers and businesses, understanding these interactions is essential to building resilient and competitive economy. As demonstrated by S1 Table, understanding how these variables interact is crucial for policymakers and businesses to develop resilient and competitive economies while balancing sustainability and economic stability.

### 2.1. The nexus between energy price and economic implications

The relationship between energy prices and economic performance indicators such as economic growth unemployment (unemployment rate, economic activity rate), inflation well-documented, highlighting the diverse impacts of energy price fluctuations on different countries’ economies. Changes in energy prices affect output, inflation, trade balances, and investment [38–40]. Studies or seminal papers have extensively examined the link between energy prices, climate regulations, and technological innovation, outlining their role in economic growth. A comparative overview of these factors across selected EU countries is presented in S1 Table. For example, countries like Germany and Poland have experienced sharp price increases due to their energy mix and carbon pricing exposure, whereas France and Norway have maintained relatively stable prices due to nuclear or hydropower dominance [41,42].

Energy price fluctuations have differing implications: while high prices can stimulate green investment, they may also dampen consumer demand and slow growth in energy-importing nations [43,44]. High energy prices can slow economic growth in energy-importing countries by increasing production costs and consumer spending [38]. The Netherlands employs demand-driven policy instruments aimed at stabilizing energy costs while promoting efficiency [45]. Norway’s integration with European energy markets has helped maintain long-term price stability despite broader regional volatility [46]. Additionally, Norway’s hydro-based energy system and high green energy production have contributed to long-term price stability and investment in clean sector [47].

### 2.2. Association between technological innovations and economic implications

Technological innovations drive economic growth, improve productivity, and enhance quality of life [48–51]. Green technologies foster productivity and offer competitive advantages, with regulations often serving as catalyst for further technological advancements [48,52]. As reflected in S1 Table, countries like Germany and Denmark lead in smart grid and wind technologies [53], while Italy and The Netherlands invest in energy efficiency and hydrogen [45,54]. Innovations are also shaped by regulation and market demand [49,55–57]. Norway’s investment in electric vehicle infrastructure has accelerated clean transport adoption, reducing emissions and dependence on imported fuels [58]. Innovations improve efficiency, create new markets, and drive demand, fostering growth in IT, biotechnology, and renewable energy while generating jobs [59,60]. Though skill shifts may lead to job losses in traditional sectors, new opportunities arise in emerging industries. Automation and IT advancements further boost global competitiveness by reducing costs and enhancing supply chain efficiency [61]. Renewable energy innovations, like solar and wind power, help reduce emissions and reduce fossil fuels dependency [62–64].

Despite challenges in adapting to market changes and skill demands, technological innovations strengthen economic resilience and support structural transformation, outweighing short-term disruptions.

### 2.3. Relationship between climate regulations and economic implications

Climate regulations shape economic activity, business strategies, and consumer behavior [65,66]. Governments worldwide are working to establish mature environmental governance systems due to the critical importance of climate regulations [67]. These regulations not only attract governmental attention but also scholarly research, exploring their impacts on innovation, competitive advantage, financial performance, and productivity [68,69]. Green economic efficiency is key to balancing environmental sustainability and economic growth [70,71]. Regulations force companies to invest in cleaner technologies, increasing costs for equipment, production changes, and emissions compliance, often passed on to consumers. As seen in S1 Table, countries such as Sweden and Denmark have adopted highly stringent regulatory frameworks, including CO₂ taxation and legally binding net-zero targets. These approaches are associated with both low emissions and innovation-driven economic growth. In contrast, countries like Belgium and Poland are still transitioning toward stricter climate policies and face challenges in reducing greenhouse gas emissions [31,72,73]. Norway’s climate strategy leverages both domestic and EU-driven incentives to advance electrification and low-carbon transitions [74].

Consumer behavior is influenced through incentives for sustainable products, such as electric vehicles. On a macro level, green policies shift employment and investment, with some industries losing jobs while green technology sectors expand. Government investment in green infrastructure further boosts private investment and job creation. In the global market, companies must balance compliance and competitiveness under varying regulatory standards. While stricter policies raise costs, international agreements help harmonize standards and facilitate trade. Nevertheless, the economic impacts of climate regulation vary significantly across national contexts. For example, the transitional costs in carbon-intensive economies can be higher in the short term, raising concerns about social and regional disparities [36].

### 2.4. The relationship between energy prices, climate regulations and innovations

These three relationships between energy prices, climate regulations, and innovations are complex and interdependent. For instance, climate regulations may increase short-term energy costs but stimulate innovation, which in turn may lower prices over time. Supplementary Material S1 Table outlines these relationships highlighting the intricate interplay between energy prices, climate regulations, and innovations, with each factor influencing and being influenced by the others in a dynamic manner [75–79]. Germany and Denmark have high energy prices but also strong renewable innovation and R&D activity [30,80]. European countries vary in their technological approaches. Germany leads in solar and wind energy, France’s nuclear power dominance helps stabilize prices but slows renewable innovation [29], Denmark enforces strict emissions targets, and Norway advances in hydropower and EV infrastructure. Spain’s solar innovation has contributed to reduced import dependency and grid decentralization [81]. Moreover, regional tariff reforms in Spain have reshaped energy market behavior and consumer participation [82]. Despite short-term adaptation challenges, technological advancements drive sustainable economic growth. Understanding the interdependence of energy prices, regulatory stringency, and technological progress is essential for crafting balanced policies that ensure competitiveness, sustainability, and long-term economic stability across the EU.

## 3. Materials and methods

The study examines relationships between climate regulations, electricity prices, and technological innovation, and macroeconomic outcomes such as inflation, unemployment, and economic growth across selected EU countries. To explore these relationships, we adopted a two-step methodology: (1) causal mapping using the Decision Making Trial and Evaluation Laboratory (DEMATEL) method and (2) statistical modeling using panel data econometrics. To gather these indicators, which express European Energy Policy, the authors utilized dataset publicly from the EUROSTAT, covering 10 European countries from 2012 to 2022. The authors selected this timeframe because the data for the chosen variables were fully available and reliable up until 2024. The selected indicators include:

- climate regulation represented by greenhouse gas emissions (GH),
- electric energy price represented by average electricity prices for non-household consumers (EP),
- technological innovations measured by research and development expenditure (RD),
- macroeconomic outcomes which are unemployment rate (UE) represented by total unemployment rate (UE), economic growth measured by gross domestic product (GDP) and inflation represented by harmonized index of consumer prices (HICP).

These indicators were selected based on a review of prior literature and their relevance to EU energy and economic policy. In this study, climate regulation is proxied by total national GHG emissions (CO₂-equivalent). While emissions are not a direct legal measure of regulatory stringency, they offer a meaningful proxy for the *de facto* effectiveness of implemented climate policies and their impact on national emissions trajectories. This approach is consistent with prior studies focusing on the environmental outcomes of regulatory frameworks. Technological innovation is proxied by total R&D expenditure as a percentage of GDP. This indicator reflects a country’s financial commitment to research and development and is commonly used in international comparisons as a proxy for innovative potential and technological progress. To identify the structure of influence among variables, we applied the DEMATEL method, which was selected due to its capacity to capture complex interdependencies among variables and to distinguish between cause and effect components within a system [83]. This technique allows modeling of complex cause-effect relationships using expert assessments. The DEMATEL analysis was conducted using a freely available online application (accessible at onlineoutput.com). This tool facilitated the construction of direct, normalized, and total relation matrices based on expert evaluations. The interface also enabled the application of a threshold value to filter significant cause-effect relationships and to generate visual outputs, such as influence maps and causal diagrams. Four selected experts from academia, policy, and industry rated the influence of each factor on the others using a pairwise scale.

Additionally, to verify the strength and direction of causal relationships, we constructed panel econometric models using open–source statistical software GRETL. It provided the computational tools necessary for Ordinary Least Squares (OLS), Fixed Effects (FE), and Weighted Least Squares (WLS) modeling, along with appropriate statistical tests such as Hausman, Wooldridge, and Breusch–Pagan diagnostics. We applied the Autoregressive Distributed Lag (ADL) approach, which accounts for both current and lagged effects of independent variables. The ADL model is a common econometric method used to capture dynamic relationships over time. It incorporates both current and past values (lags) of explanatory variables to estimate how long-term trends and short-term fluctuations jointly affect a given outcome.

**Fig 1:**
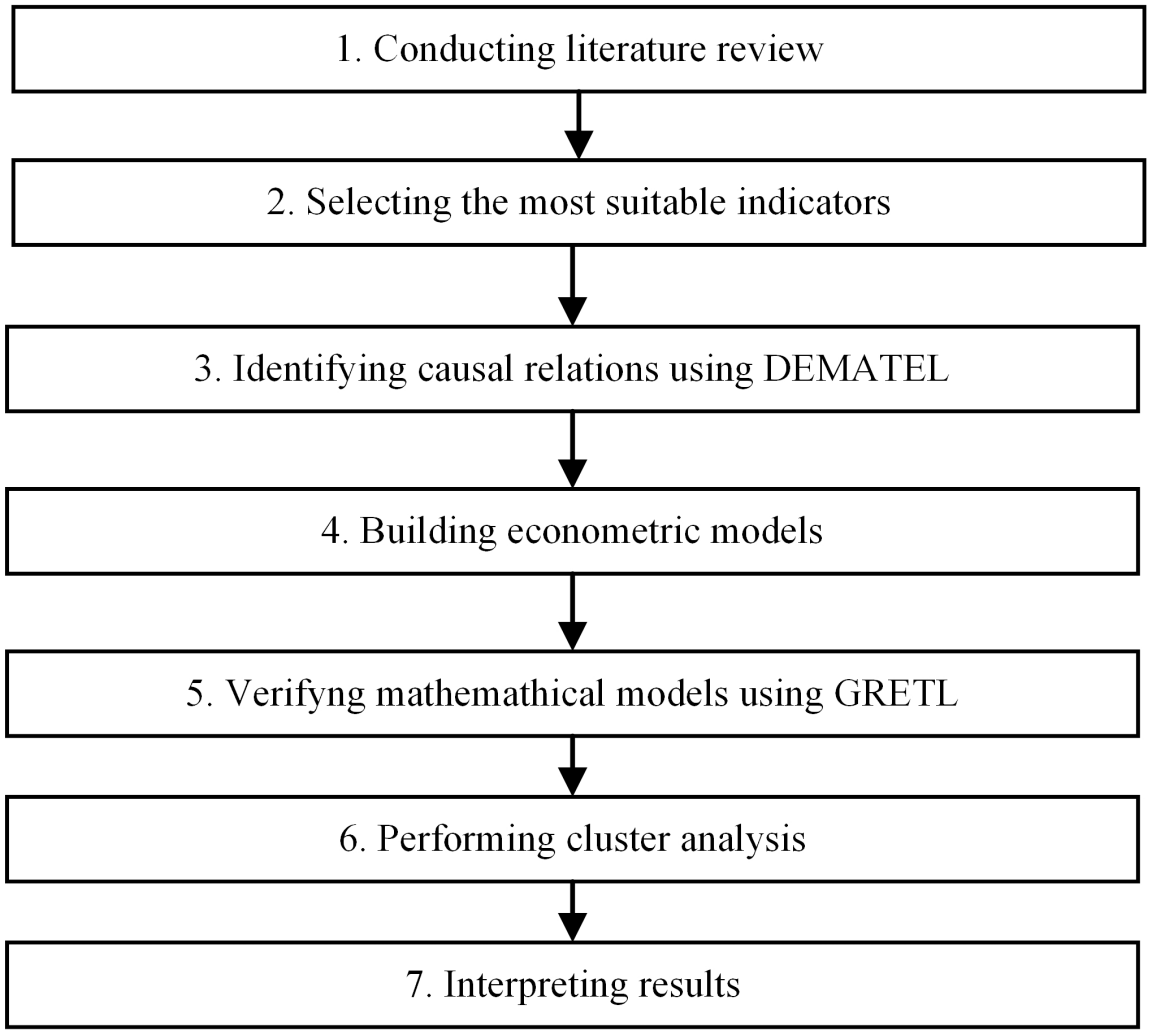
Research methodology.

Moreover, data clustering method of unsupervised learning of statistical classification was used. It is a method that groups elements into relatively homogeneous classes. The basis of grouping in most algorithms is the similarity between elements – expressed using a similarity function (metric) [84]. Grouping was used to justify the choice of the analyzed countries. This method design ensures both interpretability of causal pathways and robustness of statistical findings. The proposed research framework is depicted in Fig 1.

1. *Conducting literature review.* The study employed an analysis of existing literature and a desk research approach utilizing the researchers’ custom analytical tools tailored specifically for this analysis. Previous literature demonstrates that the evidence regarding the relationship among trade, growth, and energy is varied and inconclusive in terms of causality. Thus, the authors will attempt to investigate the existence of relationships among climate regulation, electric energy price, and technological innovations as well as unemployment rate, economic growth, and inflation. In this research, the primary data source for the empirical study was deemed to be the analysis of official documents (policies, regulations, reports, scientific papers) available on the websites of the considered countries.
2. *Selecting the most suitable indicators*. Firstly, the most suitable indicators for finding relations were identified through a review of existing literature (step 1) and presented in a theoretical model to be verified in the next step.
3. *Identifying causal analysis using DEMATEL*. To identify the structure of causal relationships among the selected indicators, we applied the DEMATEL method [83]. This method also offers robustness against subjective bias, as expert evaluations are aggregated using arithmetic means, ensuring transparency and consistency in the resulting matrices [85,86]. Compared to alternative approaches like AHP, ANP etc. [87], DEMATEL is favored for its clarity and interpretability, especially in policy contexts where decision-makers seek actionable causal insight without excessive mathematical complexity.

To conduct the DEMATEL analysis, a panel of four experts was assembled to evaluate the cause-effect relationships among key indicators. The four experts were intentionally chosen to ensure diversity and a balanced representation of academia, policy, and industry, reflecting the multidimensional nature of energy policy, innovation, and macroeconomic outcomes. The selection prioritized the depth of specialization and practical experience in the intersection of energy economics, climate regulation, and innovation systems. Although the literature often recommends 4–12 experts for DEMATEL [88], but with deep knowledge in the relevant areas for enhanced reliability, we adopted a Delphi-inspired aggregation approach to achieve consensus [89], emphasizing expert diversity over quantity. This aligns with the statement that expert diversity is more critical than sheer quantity for reliable outcomes [90]. Moreover, our study mitigates the limitations of a small panel by complementing DEMATEL results with panel econometric models, which offer independent, data-driven verification of causal relationships. This dual-method approach was designed to avoid bias stemming from expert subjectivity alone. Therefore, the panel included four experts (Table 1):

**Table 1:** Experts’ assessment concerning relations between key indicators.

Expert Code	Field of Expertise	Sector (Academia/Policy/Industry)	Years of experience
EXP01	Energy policy, Sustainability	Academia	12 years
EXP02	Socio-economy modeling	Public institution, Policy	15 years
EXP03	energy transition and socio-economic aspects	Public institution	8 years
EXP04	Energy audits and markets	Industry	15 years

Source: own elaboration.

− A university researcher specializing in sustainable energy transitions and innovation policy,
− a senior analyst dealing with public policies, including energy transition and socio-economic aspects, from a public research institute,
− an expert in economic modeling from a public research institute,
− and an industrial practitioner with experience in conducting energy audits (electricity pricing and energy market operations).

Each expert independently assessed the influence of each factor on others using pairwise comparisons. To aggregate the opinions and mitigate individual biases, the arithmetic mean was used, consistent with standard DEMATEL methodology. This approach also functions as a mechanism to manage divergent expert judgments, as averaging pairwise scores smooths extreme or outlier values and ensures that no single expert disproportionately influences the final outcome. In this way, conflicting views were harmonized through an aggregation strategy process that provides a unified and balanced consensus matrix, as highlighted in [91]. A threshold value was then determined to eliminate insignificant relationships in the total relation matrix. As recommended in DEMATEL literature [87,92], this threshold was calculated using the average of all elements in the total relation matrix (1):


$$\alpha \;=\;(\text{1}/\text{ n}²)\;\times \;\Sigma_{\text{i}=\text{1}}^{\text{n}}\Sigma_{\text{j}=\text{1}}^{\text{n}}\;{\text{t}}_{\text{ij}}$$ (1)

where tᵢⱼ represents the elements of the total relation matrix and n is the number of factors analyzed. All values below this threshold were set to zero, ensuring that only themost relevant interactions were visualized in the final cause-effect diagram.

This expert-based phase was designed with attention to methodological rigor, in line with best practices in DEMATEL research, and includes explicit procedures to address expert subjectivity, sample size constraints, and inter-rater consistency.

Some steps involved in the DEMATEL method are outlined in Table 2 as follows:

**Table 2:** DEMATEL methods’ steps.

	Purpose	Description	Formula
Step 1: Generating direct relation matrix	Quantify direct influence among factors	An n × n matrix is created to show how each factor influences the others. Expert evaluations are aggregated using the arithmetic mean.	$\text{X }=\;[{\text{x}}_{\text{ij}}]\text{ where }{\text{x}}_{\text{ij}}\;\neq \;\text{0}\text{ if i}\;\neq \;\text{j}$ (2)
Step 2: Computing the normalized matrix	Standardize scale for comparability	Normalize the direct relation matrix by dividing each element by the maximum sum of rows or columns k	$\text{k}\;=\;\text{max}(\sum {\text{x}}_{\text{ij}},\;\sum {\text{x}}_{\text{ji}});\text{ N}\;=\;\text{X}/\text{ k}$ (3)
Step 3: Calculating the total relation matrix	Reveal total (direct + indirect) influence	Calculate the total relation matrix, incorporating both direct and indirect effects	lim┬(k→+∞)〖(N^1+N^2+⋯+N^k)〗 (4)
Step 4: Setting threshold value	Highlight significant relationships and reduce noise	Eliminate minor influences by applying a threshold α. Only values above the threshold are retained	T=N×(I−Nhat(−1) (5)
Step 5: Finding final output and cause-effect diagram	Identify driving and dependent factors and visualize system structure	Calculate each factor’s influence using D + R (total impact) and identify its role with D − R (net effect), based on row (D), eq. 6 and column (R) eq. 7 sums of the total relation matrix, then visualize in a cause-effect diagram	$\text{D}\;=\;\sum_{\text{j=1}}{\text{T}}_{\text{ij}};$ (6); $\text{R}\;=\;\sum_{\text{i=1}}\;{\text{T}}_{\text{ij}};$ (7)

Source: own elaboration.

1. *Building econometric models:* in analyzing the relationships between the variables, Lagged Dependent Variables (LDV) models were used. This paper employs the autoregressive distributed lag (ADL) model, which is particularly prominent in economics and is considered the workhorse of time series models. The ADL model is relatively simple and is typically represented in the following form (8):

$${\text{Y}}_{\text{t}}\;=\alpha_{\text{1}}{\text{Y}}_{\text{t}-\text{1}}\;+{\;\beta }_{\text{0}}{\text{X}}_{\text{t}}\;+\;\beta_{\text{1}}{\text{X}}_{\text{t}-\text{1}}\;+\;\epsilon_{\text{t}}$$ (8)

where:

${\text{Y}}_{\text{t}}$–dependent variable,

${\text{Y}}_{\text{t}-\text{1}}$– dependent variable with lagged by one period,

${\text{X}}_{\text{t}}$– explanatory variable,

${\text{X}}_{\text{t}-\text{1}}$– explanatory variable with lagged by one period,

$\epsilon_{\text{t}}$– the error term.

1. *Verification of mathematical model using GRETL:* Estimation using the classical method of least squares (Pooled OLS) of the panel model is carried out using the formula (9) [93].

$${\text{y}}_{\text{it}}={\text{x}}_{\text{it}}\beta +{\text{v}}_{\text{it}}$$ (9)

where:

${\text{y}}_{\text{it}}$– the dependent variable,

${\text{x}}_{\text{it}}$–the explanatory variable (generally a variable vector of explanatory variables)

β–wektor o wymiarze N parametrów strukturalnych modelu,

${\text{v}}_{\text{it}}$–a total random error, consisting of a purely random part of $\epsilon_{\text{it}}$and an individual effect ${\text{u}}_{\text{i}}$ relating to a specific i-th unit of the panel (${\text{v}}_{\text{it}}=\epsilon_{\text{it}}+{\text{u}}_{\text{i}}$)

In the OLS estimation of the panel model, it is assumed that the index i = 1,...,N is used to denote subsequent objects, while the index t = 1,...,T is used to denote subsequent objects. Estimation using OLS is acceptable when the individual effect does not occur, and the panel is treated as a cross-sectional data set [93].

Pooled OLS is a method used in panel data analysis where data from multiple individuals or entities are pooled together and analyzed as one large sample. In Pooled OLS, all individuals are treated as a single group, and the model does not account for individual-specific effects or time-specific effects.

Another method that can be used on panel data is a panel model with (fixed effects FE) in the form eq. 10:


$${\text{y}}_{\text{it}}={\text{x}}_{\text{it}}\beta +{\text{u}}_{\text{i}}+{\epsilon_{\text{it}}}^{\text{3}}$$ (10)

where:

${\text{u}}_{\text{i}}$- the unobservable individual-specific effect;

$\epsilon_{\text{it}}$- the remainder disturbance.

In a fixed effects panel model, fixed individual effects are eliminated by averaging the model over time (t-index). Fixed Effects model is another approach in panel data analysis that accounts for individual-specific effects that are constant over time. It controls for individual heterogeneity by including individual-specific fixed effects in the model. This allows for the estimation of the relationship between variables within individuals over time.

Another model that can be used in analyses is the panel model with variable effects (random effects RE). In this type of model, individual effects ${\text{u}}_{\text{i}}$ are assumed to be a random variable. A combined random error, consisting of an individual effect and a pure random error ${\text{v}}_{\text{it}}=\epsilon_{\text{it}}+{\text{u}}_{\text{i}}$, is characterized by a correlation in the same object, while no correlation is assumed for different objects. In this situation, it is necessary to use the generalized least squares estimator of structural parameters ${\hat{\beta }}_{\text{RE}}$ of the form eq. 11:


$${\hat{\beta }}_{\text{RE}}={\left({\text{X}}^{\text{T}}\Omega^{-\text{1}}\text{X}\right)}^{-\text{1}}{\text{X}}^{\text{T}}\Omega^{-\text{1}}\text{y}$$ (11)

where:

X– the matrix of explanatory variables,

y– the vector of dependent variables

Ω– the Invertible Matrix of Variance and Covariance of Combined Random Error.

Another model is Weighted Least Squares (WLS). WLS is a modified version of OLS that accounts for heteroskedasticity by adjusting the estimation process. Unlike OLS, which assumes constant variance, WLS assigns more weight to observations with smaller variances and less to those with larger variances, reducing the impact of outliers. In panel regression with fixed effects, WLS offers several benefits: it considers spatial effects and data heterogeneity, improving model fit and accuracy. WLS also captures data heteroscedasticity, essential for accurately estimating regressor effects in models like the expectable regression with fixed effects (ERFE). Overall, WLS enhances model performance by addressing spatial effects and heteroscedasticity.

6 Performing cluster analysis

Cluster analysis is a common exploratory technique used to group data into clusters, where elements within a group are as similar as possible and different from those in other groups. Similarity is typically measured using Euclidean distance, especially for interval or ratio scale data.

In this study, the Ward’s hierarchical method was selected due to its effectiveness in minimizing intra-cluster variance using an analysis of variance (ANOVA) approach. At each step, the algorithm merges the two clusters that result in the smallest increase in total variance.

## 4. Results and discussion

### 4.1. Identifying the most suitable indicators

Based on a comprehensive literature review, the selection of indicators ensures their relevance and impact on the analysis of relationships. The chosen indicators such as electric energy price, unemployment rate, economic growth, inflation, climate regulation, and technological innovations are commonly cited in the literature for their significant influence on the dynamics of energy prices, economic performance, and environmental regulations. These indicators were selected for their recurrent presence and importance in existing literature, providing a robust framework for analyzing the interplay between energy prices, economic performance, and environmental policies.

### 4.2. Identifying causal relations using DEMATEL

Based on a comprehensive literature review, the DEMATEL method was applied to analyze the causal relationships between variables influencing inflation, unemployment, and economic growth. Due to space limitations, the full step-by-step procedure is presented for the inflation system only, while results for all three systems are summarized and interpreted. Tables 4, 6 8 present the DEMATEL results for the “Inflation”. “Unemployment” and “Economic growth” decision system. Each row shows the influence of one factor on another, with values separated by methodological steps: Step 1 – Direct Relation, Step 2 – Normalized Matrix, Step 3 – Total Relation Matrix, and Step 4 – Threshold Matrix (values below α are set to zero and are thus not considered as significant).

**Table 3:** Relation matrix – inflation.

From → To	Step 1: Direct	Step 2: Normalized	Step 3: Total	Step 4: Threshold
Energy Price → Climate Regulations	3	0.343	1.663	0
Energy Price → Technological Innovation	2.5	0.286	2.128	2.128
Energy Price → Inflation	3.0	0.343	2.012	2.012
Climate Regulations → Energy Price	2.25	0.257	1.604	0
Climate Regulations → Technological Innovation	2.5	0.286	1.569	0
Climate Regulations → Inflation	0.75	0.086	1.349	0
Technological Innovation → Energy Price	3.5	0.4	2.269	2.269
Technological Innovation → Climate Regulations	1	0.114	1.526	0
Technological Innovation → Inflation	3.5	0.4	2.075	2.075
Inflation → Energy Price	3.0	0.343	2.246	2.246
Inflation → Climate regulation	1.75	0.2	1.581	0
Inflation → Technological Innovation	3.5	0.4	2.209	2.209

Source: own elaboration.

**Table 4:** The final output – inflation.

	R	D	D + R	D-R
energy price	8.089	7.772	15.861	−0.317
climate regulations	5.769	5.522	11.291	−0.247
technological innovation	7.821	7.784	15.605	−0.036
inflation	7.225	7.826	15.05	0.601

Source: own elaboration.

Legend: Deriving cause-effect results through D + R and D − R, where: D is the sum of influences given by a factor (row sum), R is the sum of influences received by a factor (column sum), D + R shows importance, and D − R indicates causal direction (positive = cause, negative = effect).

For clarity, the threshold value used in Step 4 was α = 1.806. All values in the total relation matrix below this threshold were set to zero. The remaining values are presented in Table 3, column 4. In the inflation system, energy prices and technological innovation were identified as key influencers, while inflation itself was more of a reactive variable. The D + R and D − R values were computed to assess each factor’s total involvement and causal role. The final step involves calculating the row sums (D) and column sums (R) to assess each factor’s total importance (D + R) and net effect (D − R), as shown in Table 4.

Among the variables examined in the inflation decision system, energy prices emerged as the most systemically involved factor, exerting strong influence over both technological innovation and inflation itself (D + R = 15.861; D − R = −0.317). Technological innovation also showed a high level of interconnectedness (D + R = 15.605), positioned as a key intermediary—affected by energy prices and subsequently shaping inflationary outcomes. Inflation, in turn, occupies a dual role in the system: it both transmits and absorbs influence (D + R = 15.050; D − R = 0.601), reflecting feedback dynamics. Although climate regulations demonstrated relatively lower overall importance (D + R = 11.291) and a net reactive character (D − R = −0.247), they still contribute meaningfully to the structure of interactions. As illustrated in Supplementary Material S2 Fig 1, the results confirm that energy prices and innovation are central to inflation dynamics, while regulations, though less dominant, still shape the broader causal network.

In the unemployment decision system, the analysis (Tables 5 and 6) highlights climate regulations and energy prices as the main causal drivers. Climate regulations have the highest systemic importance (D + R = 3.976) and the strongest net causal effect (D − R = 1.169), exerting influence on energy prices, technological innovation, and the unemployment rate itself. Energy prices also play a meaningful causal role (D − R = 0.185), especially by shaping innovation and labor market outcomes.

**Table 5:** Relation matrix – unemployment.

From → To	Step 1: Direct	Step 2: Normalized	Step 3: Total	Step 4: Threshold
Energy Price → Climate Regulations	2.0	0.19	0.42	0
Energy Price → Technological Innovation	2.0	0.19	0.481	0.481
Energy Price → Unemployment Rate	3.5	0.333	0.703	0.703
Climate Regulations → Energy Price	3.5	0.333	0.681	0.681
Climate Regulations → Technological Innovation	3.25	0.31	0.672	0
Climate Regulations → Unemployment Rate	3.75	0.375	0.869	0.869
Technological Innovation → Energy Prices	2	0.19	0.423	0
Technological Innovation → Climate regulation	1.5	0.143	0.342	0
Technological Innovation → Unemployment Rate	2.5	0.238	0.565	0.565
Unemployment Rate → Energy Price	1.25	0.119	0.314	0
Unemployment Rate → Climate Regulations	1.5	0.143	0.292	0
Unemployment Rate → Technological Innovation	1.5	0.143	0.334	0

Source: own elaboration.

**Table 6:** The final output – unemployment.

	R	D	D + R	D-R
energy prices	1.733	1.918	3.651	0.185
climate regulations	1.403	2.572	3.976	1.169
technological innovation	1.754	1.597	3.351	−0.157
unemployment rate	2.425	1.229	3.654	−1.196

Source: own elaboration.

**Fig 2:**
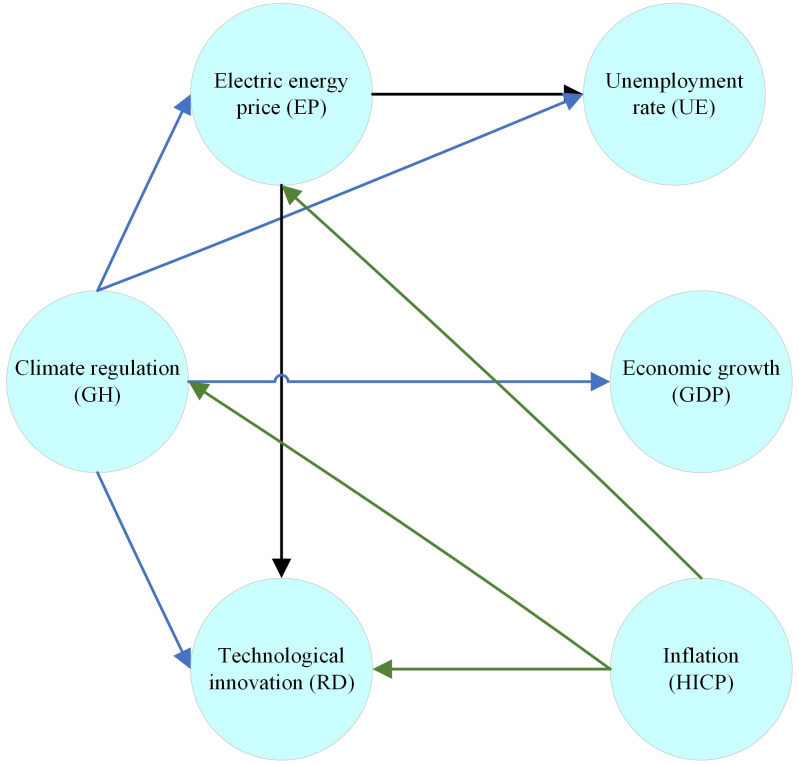
Relationships between variables.

Conversely, the unemployment rate functions primarily as a reactive variable, with a negative net effect (D − R = −1.196), indicating it is more affected by external drivers than influencing them. Technological innovation acts as an intermediary factor with moderate involvement (D + R = 3.351; D − R = −0.157), partially shaped by regulatory and energy-related changes. These interrelations are visualized in S2 Fig 2, offering insights into how regulatory frameworks and energy markets impact employment dynamics.

In the economic growth system, a threshold value of 0.593 was used to filter insignificant causal relationships from the total relation matrix. Only values above this cutoff were retained for interpretation (Tables 7 and 8).

**Table 7:** Relation matrix – economic growth.

From → To	Step 1: Direct	Step 2: Normalized	Step 3: Total	Step 4: Threshold
Energy Price → Climate Regulations	0.75	0.088	0.202	0
Energy Price → Technological Innovation	2.75	0.324	0.753	0.753
Energy Price → Economic Growth	2.5	0.294	0.827	0.827
Climate Regulations → Energy Price	3.25	0.382	0.961	0.961
Climate Regulations → Technological Innovation	3.5	0.412	1.032	1.032
Climate Regulations → Economic Growth	1.75	0.206	1.01	1.01
Technological Innovation → Energy Price	2.75	0.324	0.716	0.716
Technological Innovation → Climate Regulations	0	0	0.147	0
Technological Innovation → Economic Growth	4.0	0.471	0.955	0.955
Economic Growth → Energy Price	1.5	0.176	0.512	0
Economic Growth → Climate Regulations	0.75	0.088	0.174	0
Economic Growth → Technological Innovation	2.0	0.235	0.517	0

Source: own elaboration.

**Table 8:** The final output – economic growth.

	R	D	D + R	D-R
energy prices	2.655	2.249	4.904	−0.406
climate regulations	0.698	3.177	3.874	2.479
technological innovation	2.882	2.334	5.216	−0.548
unemployment rate	3.251	1.726	4.978	−1.525

Source: own elaboration.

The results reveal that technological innovation holds the highest total involvement (D + R = 5.216), confirming its central role within the system. However, its net causal effect is negative (D − R = −0.548), suggesting it is shaped by other variables rather than acting as a dominant driver. In contrast, climate regulations demonstrate the strongest causal influence (D − R = 2.479), despite having the lowest D + R score (3.874) among the four variables. This implies that well-established climate policies act as key initiators of systemic change.

Energy prices (D + R = 4.904; D − R = −0.406) and economic growth (GDP) (D + R = 4.978; D − R = −1.525) both function primarily as effect-type variables, receiving influence from innovation and regulations more than they contribute causally. Notably, GDP displays the most reactive behavior in the system, as evidenced by its strongly negative D − R score.

**Fig 3:**
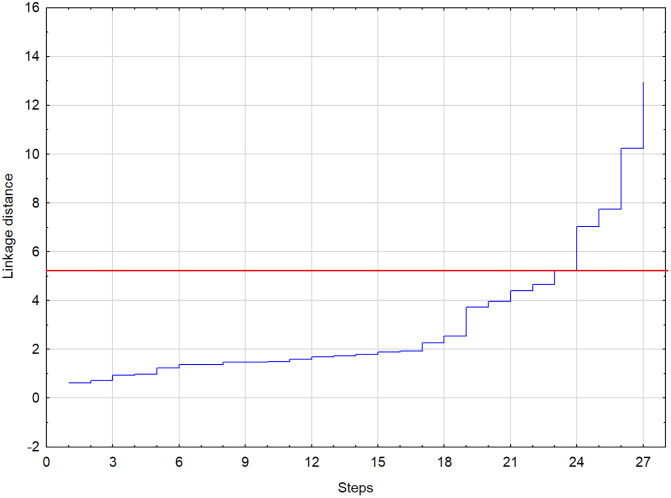
Graph of amalgamation schedule.

Together, these findings indicate that climate regulations play a pivotal causal role in shaping the conditions for technological innovation, which in turn affects downstream variables like energy prices and GDP. These dynamic underscores the importance of long-term, coherent policy frameworks for achieving sustainable economic development across EU countries. The causal structure of these relationships is visually depicted in S2 Fig 3, reinforcing the central role of regulations and the responsiveness of GDP to upstream energy and innovation policies.

### 4.3. Finding output and creating a cause-effect results

The interconnected relationships between various economic, environmental, and social indicators are illustrated in Fig 2. The relationships illustrate how changes in one indicator can cascade through to affect others, highlighting the complex interdependencies within the economic and environmental landscape.

High prices increase production costs, leading to higher unemployment rates and slowing down economic growth (GDP). They also contribute to inflation by raising the costs of goods and services. Climate regulation impacts energy prices, often increasing them due to compliance costs or shifts to renewable energy sources. Technological innovations, on the other hand, can lower energy prices through efficiency improvements. Unemployment rates are directly influenced by energy prices; higher costs lead to layoffs.

Economic growth is also tied to energy prices, with high prices reducing industrial production and consumer spending. However, technological advancements boost productivity and growth. Inflation is affected by energy prices, with higher prices driving up the costs of goods and services. Technological innovations help mitigate inflation by reducing production costs. Climate regulation impacts several indicators. It raises energy prices and affects economic growth and inflation. It also drives technological innovations as companies develop more efficient technologies to comply with regulations.

### 4.4. Verification of mathematical model using GRETL

Following the DEMATEL analysis, which helped identify how Energy Prices, Climate Regulations, and Technological Innovations influence Economic Implications across EU countries, panel data econometric models were developed. The analysis focused on the relationships shown in Fig 2, selecting only those models (Equations 12–14) that involve multiple variables. Dynamic panel models were used, incorporating lagged variables as regressors, and the estimations were performed using GRETL software. In line with standard econometric procedures [94], the estimation process began with a basic panel model using the classical Ordinary Least Squares (OLS) method, followed by a series of diagnostic tests. The Wald test indicated that a Fixed Effects Model (FEM) would be appropriate, while the Breusch-Pagan test pointed toward a Random Effects Model (REM). However, the Hausman test ultimately confirmed that the Fixed Effects Model (FEM) was the correct choice, with a 5% significance level. If heteroscedasticity is detected in further stages of the analysis, the Weighted Least Squares (WLS) method will be applied to address it. Finally, all models described by Equations 12–14 have been verified with the help of the WLS method. Analysis using DEMATEL allowed for the construction of the following relationships:


RD = f(EP, GH, HICP) (12)

UE = f(EP,GH) (13)

EP = f( GH, HICP) (14)

To capture the stochastic properties, the authors included the natural log of the variables and introduced lagged values to account for the model’s dynamic nature. The resulting models are autoregressive dynamic econometric models. For each of the models, the Pooled OLS and WLS methods were carried out. Tests performed for the models were presented in Table 9.

**Table 9:** Tests for three models from equations (12-14).

Dependent variable	Model 1 RD		Model 2 UE		Model 3 EP	
Test	Test statistic:	p-value	Test statistic:	p-value	Test statistic:	p-value
Pesaran CD test for cross-sectional dependence	z = 0.928	0.353	z = 8.972	<0.000	z = 0.530	0.596
Wooldridge test for autocorrelation in panel data	t_(9)_ = 1.061	0.316	t_(9)_ = 3.860	0.004	t_(9)_ = 1.205	0.259
White’s test for heteroskedasticity	LM = 36.489	0.493	LM = 21.461	0.370	LM = 36.489	0.493
Wald’s test for heteroskedasticity	χ^2^_(10)_ =423.941	<0.000	χ^2^_(10)_ =42.080	<0.000	χ^2^_(10)_ =606.569	<0.000
Test for normality of residual	χ^2^_(2)_ = 5.310	0.070	χ^2^_(2)_ =1.052	0.591	χ^2^_(2)_ =80.926	<0.000
Total significance of group mean inequalities	F_(9. 81)_ = 1.841	0.073	F_(9. 85)_ = 2.072	0.041	F_(9. 86)_ = 1.902	0.062

Source: own elaboration.

Model 1, based on Equation (12), is presented in Table 10. This model explores the relationship between technological innovations represented by research and development expenditure (RD) and three variables: climate regulation, measured by greenhouse gas emissions (GH); electricity prices for non-household consumers (EP); and inflation, measured by the Harmonized Index of Consumer Prices (HICP). The Weighted Least Squares (WLS) method was identified as the most appropriate estimation technique for this model.

**Table 10:** Verification of the model from equations (12-14) using WLS method.

Dependent variable	Model 1 RD	Model 2 UE	Model 3 EP
EP	−0.144***(0.023)	−0.137***(−0.040)	
EP_1	0.152***(0.030)	0.174**(−0,079)	
GH	−0.213***(0.059)	−0.432**(−0,172)	
GH_1	0.211***(0.059)	0.423**(−0,173)	0.072***(−0.015)
HICP	0.933***(0.222)		9.289***(−0.937)
HICP_1	−0.973***(0.0276)		−9.569***(−1.378)
RD_1	0.991***(0.009)		
UE_1		0.981***(−0.021)	
z711	−0.153***(0.041)		0.499**(−0.230)
z807	0.136***(0.043)		
z811			−0.732***(−0.242)
Const.	0.235(0.472)	0.200 (−0.256)	−2.278(−2.689)
No. of observations	100	100	100
No. of countries	10	10	10

Source: own elaboration.

Model 1 revealed that current electricity prices negatively impacted RD expenditure (l_EP: −0.144), while lagged prices had a positive effect (l_EP_1: 0.152). Similarly, climate regulation showed both direct (l_GH: −0.213) and lagged impacts (l_GH_1: 0.211). Inflation also significantly influenced RD expenditure, whereby immediate inflation increasing (l_HICP: 0.933) and lagged inflation decreasing (l_HICP_1: −0.9732).

Model 2 derived from equation (13) is detailed in Table 10. It investigates the relationship between the total unemployment rate (UE) and two explanatory variables: climate regulation (GH) and electricity prices for non-household consumers (EP). Prior diagnostic analysis revealed the presence of heteroscedasticity in the model’s error term. To correct this issue, the Weighted Least Squares (WLS) method was applied, ensuring more reliable and robust parameter estimation.

Model 2 demonstrated that both energy prices and climate regulations have a significant impact on employment levels. Current electricity prices were found to reduce the unemployment rate (l_EP: −0.137), whereas their lagged values were associated with an increase (l_EP_1: 0.174). Climate policy, measured by greenhouse gas emissions (GH), also affected unemployment showing a slight negative immediate effect (l_GH: −0.004) and a substantial positive lagged effect (l_GH_1: 0.423). Additionally, the lagged unemployment rate (l_UE_1: 0.981) was highly significant, indicating strong persistence in labor market dynamics.

Model 3, based on Equation (13), is presented in Table 10. This model analyzes the relationship between electricity prices for non-household consumers (EP) and two explanatory variables: climate regulation, measured by greenhouse gas emissions (GH), and inflation, measured by the Harmonized Index of Consumer Prices (HICP). As with the previous models, the Weighted Least Squares (WLS) method was determined to be the most appropriate approach for estimation.

Model 3 revealed a strong effect of lagged electricity prices on current prices, with a highly significant positive relationship (l_EP_1: 0.947). Additionally, current inflation (l_HICP: 7.161) was found to increase electricity prices, while lagged inflation had a negative impact (l_HICP_1: −6.522).

The findings of this study highlight the multifaceted impact of energy prices and climate regulations on key economic variables such as technological innovation and labor market dynamics. Both energy costs and environmental policies exhibit significant short- and long-term effects, underscoring the importance of accounting for lagged relationships in policy design. The results suggest that maintaining stable energy prices and ensuring regulatory predictability may foster investment in research and development while mitigating adverse effects on employment. Inflation emerged as a critical factor influencing all modeled relationships, indicating that macroeconomic and monetary policy considerations should be integrated into the broader energy and climate policy framework. Overall, the evidence supports a long-term, balanced approach to policymaking—one that aligns environmental objectives with economic growth and social stability.

### 4.5. Performing cluster analysis

In the next step, a cluster analysis was carried out. All members of the EU were determinedly selected for research on 11 June 2025 (27 countries – EU −27). The research period embraced the year 2023 (the latest complete data for all indicators, which could be downloaded from Eurostat). The clustering was conducted based on indicators analysed earlier, namely EP, UE, HICP, RD, GBP, GH.

**Fig 4:**
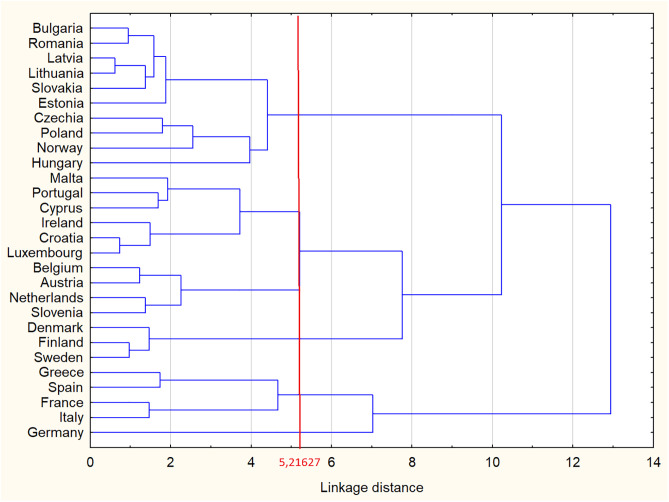
Tree diagram: hierarchical cluster analysis of six analysis indicators (EP, UE, HICP, RD, GBP, GH) in European countries in 2023.

A commonly employed and relatively straightforward method for determining the optimal number of clusters involves cutting the dendrogram at the point corresponding to the first substantial increase in agglomeration distance. At each stage of the hierarchical clustering process, a minimum distance value can be identified—this value separates the newly merged element from all remaining clusters. When the increment between two successive agglomeration distances reaches its maximum, it is considered a logical point to terminate the clustering procedure. An effective analytical tool for identifying this point is the agglomeration schedule plot. The results are given in Fig 3 and Fig 4. The tree diagram (Fig 4) is the first and the simplest result of the cluster analysis, and it is closely related to the second result, the graph of amalgamation schedule (Fig 3). The algorithm first calculates all the Euclidean distances between the countries (and puts them in the tree diagram), and only after arranging the distances in an ascending scale, it shows the amalgamation schedule.

The key to interpreting a hierarchical cluster analysis is to look at the point at which any given pair of countries “join together” in the tree diagram. Countries that join sooner are more similar to each other than those that join later. To find the optimal number of clusters, use the graph of amalgamation schedule. One could observe that at 24th step, The Euclidean distance increases rapidly by more than 2 units from a value of 5.2 to a value of 7.5 (indicated by the red line in Fig 3). Determining 5,2 as a cutoff point (as suggested by the amalgamation schedule in Fig 3 results in five distinct clusters of EU countries (indicated by red line in Fig 4).

Table 11 and Table 12 confirm the results of cluster analysis, presenting the assignment of the EU-27 countries to individual clusters and the average values of the analyzed indicators that were used for this analysis.

**Table 11:** Distribution of EU-27 countries into clusters based on six analysis indicators (EP, UE, HICP, RD, GBP, GH).

Cluster	Countries
Cluster 1	Belgium, Ireland, Croatia, Cyprus, Luxembourg, Malta, Netherlands, Austria, Portugal, Slovenia
Cluster 2	Bulgaria, Czech Republic, Estonia, Latvia, Lithuania, Hungary, Poland, Romania, Slovakia, Norway
Cluster 3	Denmark, Finland, Sweden
Cluster 4	Greece, Spain, France, Italy
Cluster 5	Germany

Source: own elaboration.

**Table 12:** Presentation of the average values of six indicators (EP, UE, HICP, RD, GBP, GH) for each cluster of EU-27 countries.

	Cluster 1	Cluster 2	Cluster 3	Cluster 4	Cluster 5
EP [€/kWh]	0.21	0.17	0.10	0.18	0.19
GDP [%]	313422	239323	399918	1 642 667	4122210
UP [%]	4.92	4.86	6.67	9.58	3.10
HICP [-]	122.99	144.06	120,9	119.28	125,90
RD [%]	1.80	1.27	3.23	1.62	3.11
GH [thousand tonnes]	47857	65755	35017	239 234	740673

Source: own elaboration.

The 1st Cluster is characterized by highest values of variable electric energy price represented by electricity prices for non-household consumers and one of the lower value averages economic growth measured by gross domestic product and average value unemployment rate. Countries belonging to the 2nd Cluster have the highest average inflation represented by harmonised index of consumer prices and the lowest average value research and development expenditure. Countries in the 3rd Cluster are characterized by the lowest average value electricity prices for non-household consumers and highest averages research and development expenditure. The 4th Cluster consists of countries with the lowest averages inflation represented by harmonised index of consumer prices and technological innovations measured by research and development while the highest value unemployment rate represented by total unemployment rate. Country belonging to the 5th Cluster has the highest averages economic growth measured by gross domestic product and climate regulation measured by greenhouse gas emissions while the lowest averages unemployment rate represented by total unemployment rate.

The cluster analyses confirm the appropriateness of the chosen countries as representatives of the broader European context, as they reflect the full spectrum of the identified clusters. By including at least one country from each cluster – Germany (Cluster 5); France, Italy, and Spain (Cluster 4); Sweden and Denmark (Cluster 3); Poland and Norway (Cluster 2); and the Netherlands and Belgium (Cluster 1), the analysis captures the diversity of economic and regulatory conditions across the European Union and associated states.

The cluster analysis was not based on a priori hypotheses but was conducted to explore the internal structure of the dataset and validate the selection of ten representative countries used throughout the study. These countries were selected based on prior literature as exemplars of different energy and economic strategies within the EU.

The clustering results confirmed this assumption, as the selected countries were distributed across all clusters and captured the spectrum of regulatory and economic diversity in Europe. While the concept of cluster theory is referenced for contextual framing, the study does not aim to test its specific theoretical postulates. Future research may integrate frameworks such as regional innovation systems or governance typologies to further interpret clustering outcomes.

### 4.6. Interpretation of counterintuitive results

Beyond these results, some relationships exhibited counterintuitive dynamics that merit further interpretation and policy contextualization. The observed counterintuitive effect of lagged inflation reducing R&D expenditure can be interpreted in the context of the cost-push inflation theory, where inflation increases input costs (e.g., wages, materials), thereby compressing corporate margins and limiting available funds for investment. This “crowding-out” effect is particularly acute in periods following high inflation when firms recalibrate spending and prioritize short-term liquidity over long-term innovation. Similar findings have been reported in studies examining inflationary uncertainty and investment restraint under fiscal stress. For instance, Lin et al. (2021) found that heightened uncertainty significantly reduces global R&D expenditure, the number of researchers, and patent applications [95]. Likewise, panel studies across G20 countries confirm that macroeconomic uncertainty dampens business R&D growth, especially during periods of economic volatility. Similar findings related to the suppression of investment under conditions of uncertainty or macroeconomic stress have been presented in studies analyzing the impact of climate-related uncertainty on investment decisions [96], as well as in research highlighting the sensitivity of innovation to financial and economic disruptions in European countries [60]. These findings support the interpretation that inflation, especially when lagged can function as an investment suppressor, as firms facing elevated costs and uncertainty reduce their forward-looking expenditures in favor of short-term liquidity.

From the DEMATEL results, several clear implications for policy emerge. In all three decision systems (inflation, unemployment, and GDP growth), climate regulations consistently act as a dominant causal driver, exerting significant influence on both energy prices and technological innovation. In contrast, energy price controls tend to function more as reactive or effect variables, particularly in the economic growth system (D − R < 0).

Therefore, the results suggest that policy efforts should prioritize the design and enforcement of coherent, long-term climate regulation frameworks, which not only steer technological advancement but also indirectly stabilize economic variables. Rather than intervening directly in price mechanisms (e.g., subsidies or price caps), governments could achieve more sustainable outcomes by making climate regulation predictable, innovation-oriented, and economically integrated. This approach would reduce uncertainty for investors, stimulate green R&D, and indirectly moderate inflationary pressures by improving energy efficiency.

Using the integrated methodological approach combining DEMATEL and panel econometric models, including autoregressive dynamic models, the study identified key and policy-relevant relationships between variables. Key findings reveal that:

− climate regulations consistently act as dominant causal drivers, influencing both technological innovation and energy prices across all decision systems,
− technological innovation is a central variable in the system, shaped by regulatory and price mechanisms and playing a crucial role in promoting economic resilience,
− energy prices, although important, largely behave as effect variables, particularly in relation to GDP growth (as shown by negative D−R values in DEMATEL).

The results of the econometric analysis show the key findings from the three regression models, highlighting the direction and timing of the relationships between the explanatory variables (electricity prices, climate regulation, inflation) and the dependent variables (R&D expenditure, unemployment, and electricity prices). The interpretation focuses on both immediate and lagged effects, offering insights into short- and long-term dynamics relevant to energy policy, innovation, and labor markets. These results are discussed in the context of established economic theories, including Porter’s Hypothesis and labor market adjustment frameworks.

Model 1 – Determinants of R&D Expenditure – presents that R&D spending is significantly influenced by electricity prices, climate regulation, and inflation. Current electricity prices negatively affect R&D, while their lagged values show a positive effect. This suggests that innovation investments tend to follow energy price increases with a delay. A similar dual mechanism is observed for climate regulations: they constraint R&D in the short term but stimulate it in the longer term, partially supporting Porter’s Hypothesis. Inflation has a positive immediate effect on R&D, possibly due to anticipatory investments but its lagged impact is negative, indicating that sustained inflation eventually restricts innovation capacity. However, in the case of Model 2 – Determinants of Unemployment – unemployment is sensitive to changes in energy prices and climate policy. Current electricity prices reduce the unemployment rate, which may be attributed to short-term stimulus in energy-intensive sectors. However, lagged prices lead to higher unemployment, reflecting delayed adjustment costs in the labor market. Climate regulation, measured through GHG emissions, has a minimal short-term effect but a strong positive lagged impact on unemployment, suggesting restructuring pressures in the economy. The highly significant lagged unemployment variable confirms the persistent and path-dependent nature of labor market dynamics in European countries. Conclusion from Model 3 – Determinants of Electricity Prices- is Electricity prices display strong temporal stability, with a highly significant positive relationship to their lagged values, indicating predictable price dynamics. Inflation is another key driver: current inflation pushes electricity prices upward, while lagged inflation shows a negative effect. This may reflect the influence of inflation-targeting policies or adaptive pricing mechanisms that respond with a delay to macroeconomic conditions.

## 5. Conclusions

### 5.1. Summary of findings

This study addressed the research gap by analyzing the national diversity in European energy policies and their relation with economic impacts. The key indicators: climate regulations (GH emissions), electricity prices (EP), and technological innovations (R&D expenditures), while economic impacts were assessed through unemployment (EU), economic growth (GDP), and inflation (HICP).

The findings clearly demonstrate the complex and dynamic nature of the interplay between energy and climate policy and macroeconomic variables such as technological innovation, inflation, and the labor market. This can be effectively linked to key European initiatives: Fit for 55, REPowerEU, and the EU ETS, which play a central role in the European Union’s energy and economic transformation. The results support the approach pursued by the EU: the energy transition must be complementary to macroeconomic goals and not implemented at the expense of employment, price stability or the development of innovation. Therefore, the coordinated policies (climate, energy, fiscal and monetary), long-term regulatory frameworks, and innovation incentives are required to manage the transition costs and stimulate adaptive capacity.

This study offers holistic perspective by comparing EU countries with different energy systems, enabling the identification of cross-national differences in policy effects and patterns of action. By using quantitative tools (DEMATEL, panel models), the article captures the linkages between energy policies and economic stability in a way that has not been sufficiently present in previous publications. Additionally, the article treats energy prices, innovation and regulation not as isolated variables but as interdependent elements influencing the economy under conditions of instability (war, inflation, technological change).

**Fig 5:**
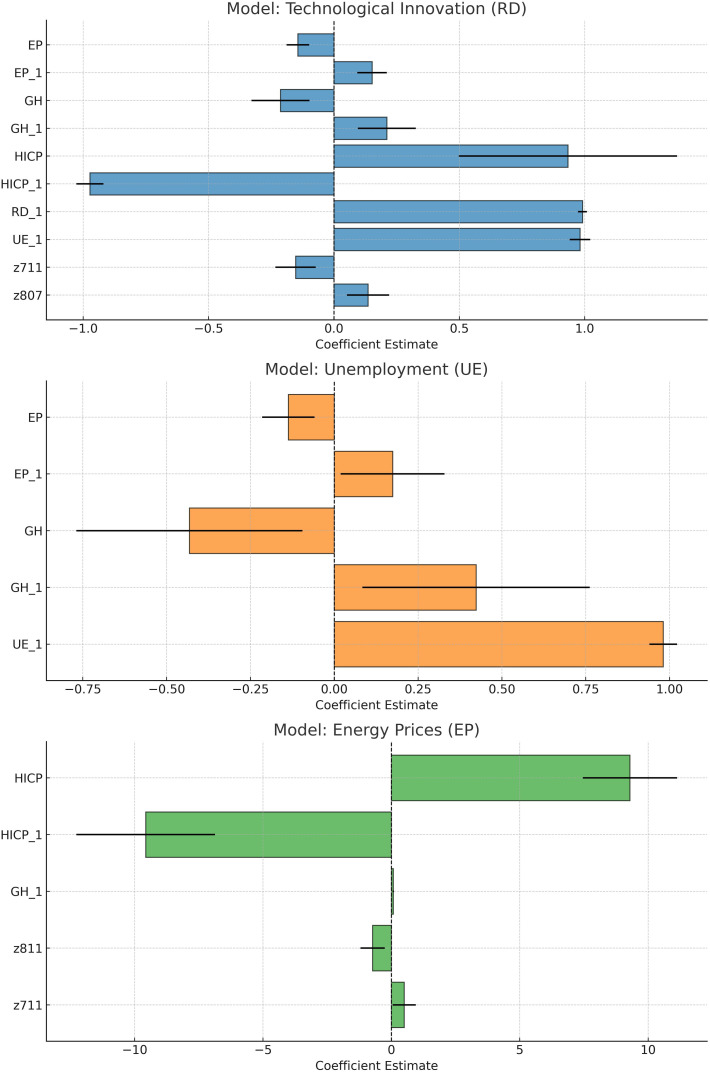
Coefficient Plots for the three models (with 95% confidence intervals).

As visualized in Fig 5 the coefficient further validates the econometric findings by confirming dynamic, time-lagged relationships across the three core models and by illustrating the direction, strength, and confidence intervals of the estimated effects. In the innovation model, inflation shows a dual effect: current inflation stimulates R&D, while lagged inflation reduces it, consistent with cost-push dynamics. Energy prices and emissions also shift from short-term negative to lagged positive effects on innovation, suggesting firm adaptation over time. In the unemployment model, energy prices and emissions initially reduce unemployment, likely via sectoral job growth, but increase it with a lag, reflecting delayed cost impacts. Unemployment itself remains highly persistent. In the energy price model, inflation dominates current inflation drives prices up, while lagged inflation aligns with later corrections, possibly from regulatory or demand adjustments. Other variables show modest or context-specific effects.

These dynamic relationships highlight the importance of incorporating temporal structure into energy and innovation policy design. They also confirm the central roles of inflation, regulatory stability, and innovation incentives in shaping economic resilience.

Together, these findings contribute both methodological and practical value. They demonstrate the relevance of integrated causal-econometric modeling for capturing time-dependent policy interactions. For policymakers, the results underscore the importance of designing forward-looking, adaptive frameworks that coordinate regulatory, energy, and economic objectives across time and sectoral contexts.

#### Empirical reflection on porter’s hypothesis.

Porter’s hypothesis assumes that appropriately designed environmental regulations – rather than hindering economic development – can stimulate business innovation and increase their competitiveness. In light of research findings indicating that climate regulations are drivers of innovation, Porter’s theoretical assumptions are empirically supported. In the context of EU climate policy, ambitious regulations on CO₂ emissions have led to the development of renewable energy technologies and innovations in transport (e.g., electromobility) – which is a practical confirmation of Porter’s hypothesis. Nevertheless, an innovative response from companies does not appear automatically – it depends on, among other things, the structure of the sector, R&D capabilities, and institutional support. Moreover, regulations must be predictable, flexible, technologically neutral and focused on efficiency, not excessive bureaucracy. This means that climate regulations not only do not restrict companies’ activities but can also act as a stimulus to pursue new technological solutions, improve the efficiency of production processes, and even create new business models. These innovations can contribute to increased productivity, reduced long-term costs, and improved competitive position of companies – both domestically and internationally. Consequently, in the context of climate policy, regulations treated as levers for innovation align with the “weak version” of Porter’s hypothesis (increased innovation), and if they also support increased productivity and profits, with its “strong version” (increased competitiveness). Thus, climate regulations can be viewed not as a cost but as a catalyst for technological transformation and sustainable economic development. These findings extend the application of Porter’s hypothesis beyond firm-level analysis, providing support for its relevance in the context of EU-wide climate regulation frameworks.

### 5.2. Policy implications

A novel contribution of this study lies in identifying the dual role of inflation in shaping R&D investment. The econometric analysis shows that current (contemporaneous) inflation stimulates R&D, possibly due to forward-looking adjustments or strategic hedging against future cost increases. In contrast, lagged inflation significantly suppresses R&D, consistent with cost-push inflation theory, where increased input costs crowd out long-term investments. This asymmetry demonstrates the time-sensitive and nonlinear nature of inflation’s influence, a dynamic largely overlooked in previous energy policy literature.

The study suggests that policy efforts should prioritize long-term, climate regulation frameworks, that actively incentivize green innovation through concrete instruments. These may include carbon contracts for difference (CCfDs) [97], R&D tax credits or sectoral R&D incentives, and green public procurement schemes [98]. Another key initiatives and climate regulations of EU are Fit for 55, REPowerEU, and EU ETS. The first one is a legislative package presented by the European Commission in July 2021, aimed at reducing greenhouse gas emissions in the EU by at least 55% by 2030 compared to 1990 levels [99]. REPowerEU is the European Commission’s May 2022 plan to address the energy crisis caused by Russia’s war against Ukraine. Its goal is to end the EU’s dependence on Russian fossil fuels by 2027 [100]. The third one is the main mechanism of the EU’s climate policy, the CO₂ emissions trading system, was introduced in 2005 and covers the largest emitters (e.g., electricity, industry, and aviation) [101]. Such tools have been shown to be more effective than direct price interventions (e.g., energy price caps or untargeted subsidies) in stimulating technological progress, reducing macroeconomic volatility, and supporting sustainable energy transitions.

However, the effectiveness of these policy tools varies across European countries and depends on institutional capacity and economic conditions, as well as the feasibility of implementation, as illustrated in Table 13.

**Table 13:** Cluster – specific policy recommendations.

Recommendation/ Cluster	Cluster 1:	Cluster 2:	Cluster 3:	Cluster 4:	Cluster 5:
Countries	Belgium, Ireland, Croatia, Cyprus, Luxembourg, Malta, Netherlands, Austria, Portugal, Slovenia	Bulgaria, Czech Republic, Estonia, Latvia, Lithuania, Hungary, Poland, Romania, Slovakia, Norway	Denmark, Finland, Sweden	Greece, Spain, France, Italy	Germany
Policy focus	Reducing energy costs for industry and innovation; stimulating economic growth	Macroeconomic stabilization; increasing investment in innovation	Continuing proinnovation policy; strengthening technological leadership and green technology exports	Increased employment and labor market activation; increased efficiency of innovation spending	Maintaining economic and climate leadership; exporting technological solutions
Recommended instruments	Subsidies or incentives for the industrial sector in support of energy transition Energy market reform (e.g., improving competitiveness, investing in renewable energy sources)	Tax relief for R&D activities; monetary policy aimed at combating inflation	Supporting private-public partnerships in green technologies; funding research, development, and technology transfer	Regional innovation and smart specialization strategies; a greater role for EU funds in stimulating demand for R&D	Investments in climate infrastructur; supporting innovating business models
Feasibility conditions	High implementation potential; strong public institutions; The social need to maintain the economy’s competitiveness in the face of high energy costs.	Moderate feasibility; needs external financing and political consensus; the need to improve the efficiency of public institutions	High feasibility; strong administrative and fiscal capacity; good experience in integrating environmental and innovation policies	High social pressure to combat unemployment; problems with administrative efficiency and bureaucracy	A strong industrial and institutional base; the need to maintain a balance between climate change and economic competitiveness

The division into clusters shows the diversity of economic and political conditions in Europe, which justifies the need for a differentiated approach to energy and climate policy. Therefore, promoting green innovation should not follow a one-size-fits-all formula, but rather adapt to the institutional and governance landscape of each cluster. Embedding innovation policy within stable, long-term regulatory frameworks — tailored to national capacity and supported by EU-level coordination remains key to enabling a credible and effective energy transition [102].

Current EU-wide measures such as eco-design standards have positive effects on energy efficiency [103], but have not yet produced measurable reduction in consumption or energy dependency [104], indicating the need for more effective strategies. As energy prices directly influence macroeconomic performance [105], adaptive, innovation-oriented policies that align with institutional capacity can improve both policy credibility and implementation feasibility.

The current selection of indicators is based on data availability, comparability, and consistency with previous literature. However, future research could improve measurement precision and the validity of conclusions by employing more complex indicators of regulation, innovation efficiency, and inflation composition. This would allow for an even more sophisticated analysis of the impact of energy and climate policies on the economy.

### 5.3. Limitations

While the findings offer valuable insights into the interaction between energy policy and economic indicators, several limitations must be considered. First, the model does not capture recent macroeconomic shocks such as the COVID-19 pandemic and the Russia – Ukraine conflict, both of which caused widespread disruption to energy systems, supply chains, and inflationary trends in Europe. Recent studies have shown how such crises altered R&D investment patterns and increased macroeconomic volatility, complicating causal inferences [106]. Such events may have altered inflation dynamics and energy price volatility, thereby affecting the robustness of the causal interpretations. Second, although panel models include fixed effects to control for country-specific heterogeneity, differences in energy infrastructure, regulatory enforcement, and institutional capacity across EU member states can introduce measurement inconsistencies [107]. Third, the analysis focuses on selected key indicators, omitting variables such as institutional quality, energy storage readiness, and consumer-level behavioral responses. Recent studies have emphasized the importance of political institutions and trust in shaping policy effectiveness [80].

The use of proxy variables, while necessary due to data availability constraints, introduces certain limitations. Greenhouse gas (GHG) emissions were employed as a proxy for climate regulation, reflecting the de facto outcomes of policy rather than its formal stringency (de jure). This approach is commonly applied in macro-level empirical studies when direct measures of policy instruments (e.g., carbon taxes or ETS coverage) are not consistently available across countries and time.

Similarly, R&D expenditure was used to represent technological innovation. While this variable captures a country’s investment in innovation, it does not reflect the efficiency or sectoral distribution of R&D efforts. Lastly, inflation was measured via the Harmonized Index of Consumer Prices (HICP), which is a standard measure but does not differentiate between core and energy-related inflation.

These limitations are acknowledged and suggest several directions for future research, including the incorporation of more nuanced indicators such as the OECD Environmental Policy Stringency Index, World Bank Governance Indicators, or disaggregated inflation metrics, where available.

These additional factors may explain part of the unexplained variance in economic or innovation outcomes under transition stress. Finally, the DEMATEL method relies on subjective expert judgments. While the aggregation approach reduces individual bias, it does not fully eliminate the influence of the expert pool’s contextual background and domain perspectives [86].

### 5.4. Future research

While the results present the cross-national view on the dynamic interplay between energy policy, innovation, and macroeconomic outcomes, several directions for further research emerge. First, future studies should enhance the precision of regulatory measurement by incorporating de jure indicators of climate policy such as the OECD Environmental Policy Stringency Index (EPS) or European Union Emissions Trading System (EU ETS) coverage metrics rather than relying solely on outcome-based proxies like GHG emissions [108,109]. Additionally, the next research could also incorporate institutional quality indicators, such as the World Bank’s Worldwide Governance Indicators (WGI). In particular, Regulatory Quality, Government Effectiveness, and Rule of Law may help explain cross-country differences in policy impact and innovation responsiveness [110]. These institutional metrics can provide context for understanding why countries with similar climate goals may differ significantly in regulatory outcomes or R&D effectiveness, thus offering a valuable lens for analyzing implementation feasibility and political economy dynamics.

Second, future studies could consider multi-dimensional innovation indicators, combining R&D expenditure with measures of R&D efficiency, as emphasized by [43], sectoral allocation, and innovation outputs (e.g., patents or green technology deployment) to further enrich policy design and strategic energy planning. Third, deepen contextual understanding and improve policy targeting, more localized data and temporally. extended datasets should be used, especially at the subnational or sectoral level. This could help distinguish short-term fluctuations from structural transitions.

Specific research questions that require further exploration include:

- How does the formal stringency of environmental regulation (e.g., carbon taxes, ETS participation) affect innovation performance across sectors and countries with varying institutional quality?
- To what extent does regulatory quality moderate the relationship between energy prices and macroeconomic volatility?
- Can composite governance and innovation indices improve the predictive power of models assessing the economic impacts of green transitions?

## Supporting information

S1 Table Overview of energy policy approaches and economic implications in selected EU countries. (DOCX)

S2 Fig Related to significant relations. S2 Fig 1 The model of significant relations – inflation. S2 Fig 2 The model of significant relations – unemployment. S2 Fig 3 The model of significant relations – economic growth.
(DOCX)
