# Supplementary material for: Uncovering economic impacts and dynamics of European energy policy: Evidence from DEMATEL, panel data, and cluster analysis
Source: PLoS One. 2025 Nov 4;20(11):e0322525. doi: 10.1371/journal.pone.0322525 (PMC12585084; doi:10.1371/journal.pone.0322525)
Supplement: S1 Table — (DOCX) [file pone.0322525.s001.docx]

**S1 File. Supplementary material**

Uncovering economic impacts and dynamics of European energy policy: Evidence from DEMATEL, panel data, and cluster analysis

**S1 Table.** Overview of energy policy approaches and economic implications in selected EU countries

| Country | Energy Policy Approach | Economic Implications | Changes in Energy Prices | Climate Regulations | Technological Innovations |
| --- | --- | --- | --- | --- | --- |
| Germany | Transition to renewables, strong regulatory framework (Fischer et al., 2016) | High investment costs, balanced inflation (Adedoyin et al., 2023; Chong, 2015) | Significant increase in industrial and household energy prices since 2019 due to carbon pricing and RES expansion | Stringent regulations aligned with EU Green Deal; strong ETS implementation | Smart grids, storage technologies |
| France | Nuclear energy dominance, gradual decarbonization (Chong, 2015) | Moderate inflation, stable GDP growth | Moderate and stable prices due to dominance of nuclear energy | Moderate regulation with gradual reforms; strong focus on electrification | Advanced nuclear tech, R&D incentives |
| Sweden | Hydro and nuclear mix, high energy efficiency | Low inflation, green GDP growth | Decreasing trend in electricity prices due to low-carbon energy surplus | Very stringent targets, including net-zero by 2045 and CO2 taxation | Carbon neutrality focus, digital grid |
| Poland | Coal-reliant, under EU transition pressure (GOV, 2021) | High inflation, moderate unemployment | Sharp increases in energy prices due to dependency on coal and EU ETS costs | Transitional climate policy; catching up with EU standards | Emerging renewable investments |
| The Netherlands | Gas and offshore wind, carbon tax adoption (Khorishko, 2021) | Moderate inflation, steady growth | Moderate increase; high energy taxes offset by renewables support | Stringent climate law with carbon pricing and offshore wind targets | Green hydrogen, offshore wind (IEA, 2020) |
| Spain | Liberalized market, solar expansion | Recovery phase, regional disparity | Volatile energy prices due to market liberalization and fuel cost fluctuations | Moderate regulation; incentives for solar and efficiency | Solar R&D, smart metering (Espinosa and Pizarro-Irizar, 2018; Manso-Burgos et al., 2021) |
| Denmark | Wind energy leadership, market incentives (Lund et al., 2022; Sovacool, 2013) | Stable economy, high R&D ratio | Relatively stable prices, supported by large wind share | Strong regulation with binding targets for wind and electrification | Wind innovation, digitalization |
| Belgium | Nuclear phase-out in progress (Soytas et al., 2022; Nijs and Regemorter, 2012) | Supply uncertainty, moderate growth | Gradual rise in prices amid nuclear transition and market pressure | Moderate regulation with nuclear exit strategy and RES support | Grid modernization, renewables |
| Italy | Diversified mix, efficiency programs (Caragliu, 2021) | Moderate inflation, innovation-driven | Rising prices with volatility due to import dependence | Moderate regulation, focus on energy efficiency and consumption control | Energy storage, building retrofits |
| Norway | Hydropower surplus, export-oriented (Austvik, 2019; Malka et al., 2023) | Stable prices, high investment rate | Stable prices due to hydropower dominance; linked to European market | Stringent targets and electrification policies; low emissions baseline | Green transport, electrification (Jåstad et al., 2022; Skjærseth and Jevnaker, 2018; Zhou et al., 2022) |

Note: References cited in this table are included in the general reference list
