# Supplementary material for: Uncovering economic impacts and dynamics of European energy policy: Evidence from DEMATEL, panel data, and cluster analysis
Source: PLoS One. 2025 Nov 4;20(11):e0322525. doi: 10.1371/journal.pone.0322525 (PMC12585084; doi:10.1371/journal.pone.0322525)
Supplement: S2 Fig — 1 The model of significant relations – inflation. S2 Fig 2 The model of significant relations – unemployment. S2 Fig 3 The model of significant relations – economic growth. (DOCX) [file pone.0322525.s002.docx]

**S2 File. Supplementary material**

Uncovering economic impacts and dynamics of European energy policy: Evidence from DEMATEL, panel data, and cluster analysis


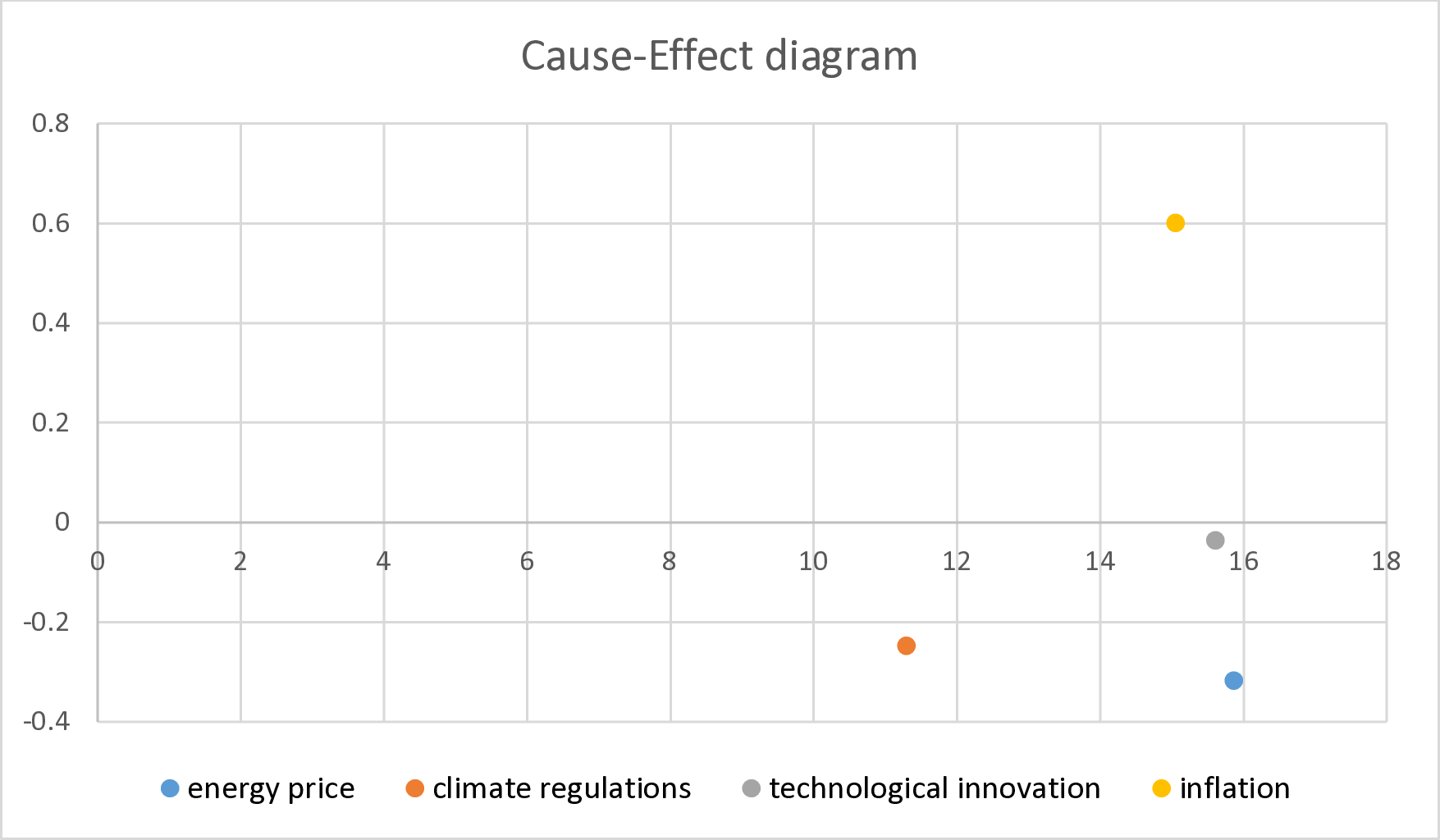


**S2 Fig. 1** The model of significant relations- inflation


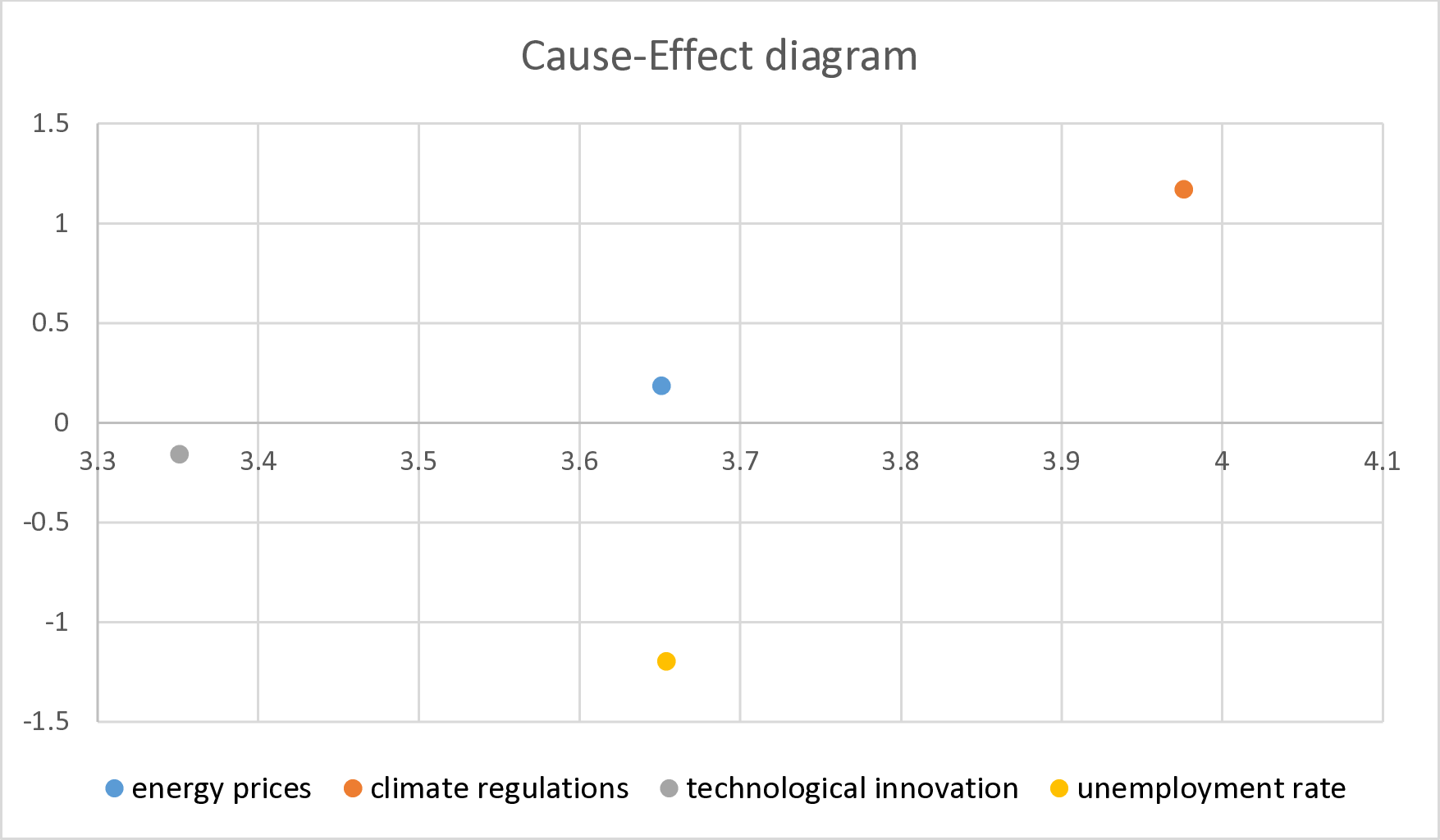


**S2 Fig. 2** The model of significant relations - unemployment


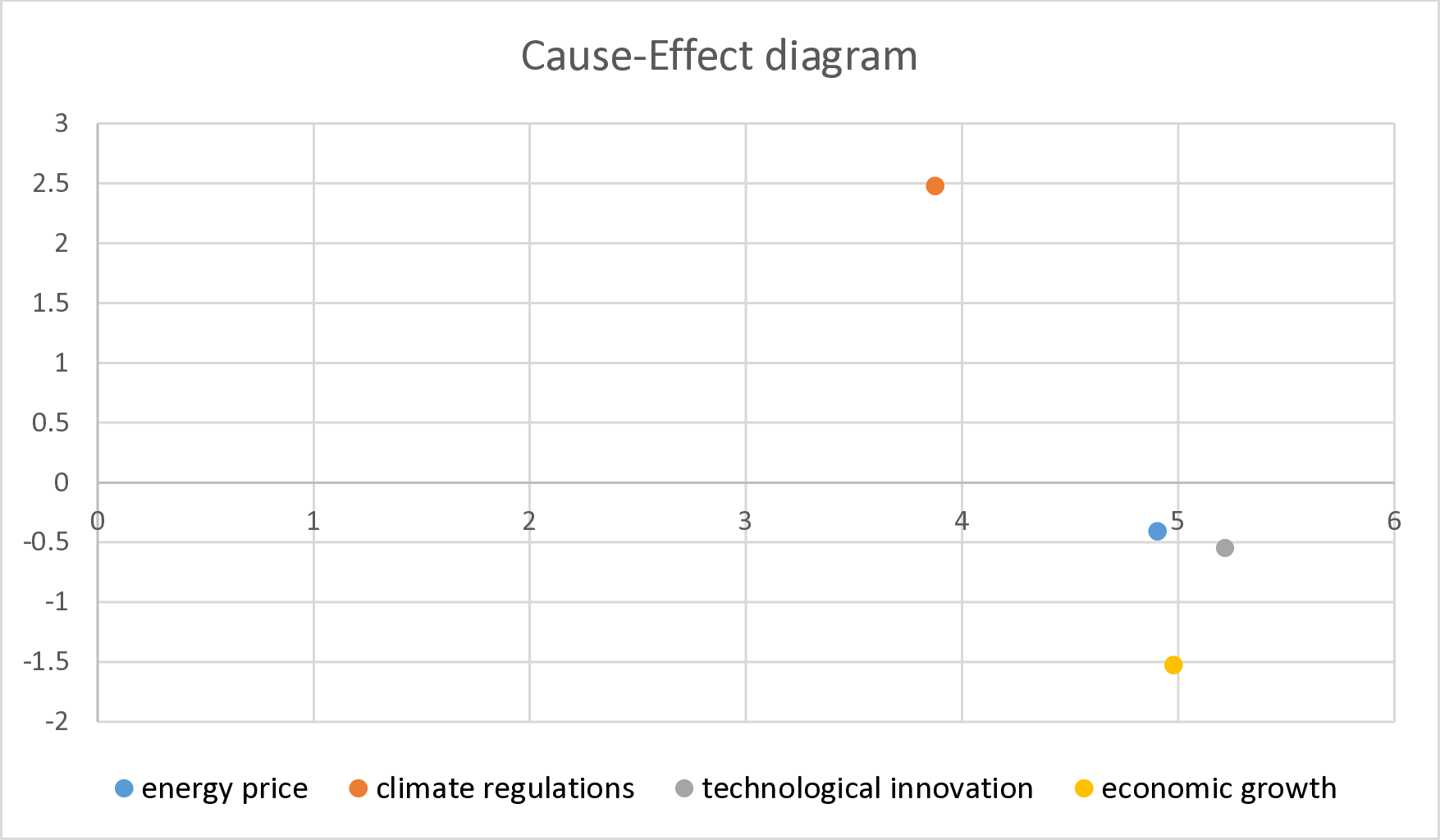


**S2 Fig. 3** The model of significant relations – economic growth
